# Psycho-Behavioral Characteristics Perceived as Facilitators by Brazilian Adults with Type 1 Diabetes Mellitus in a Public Health Service

**DOI:** 10.3390/healthcare11162300

**Published:** 2023-08-15

**Authors:** Priscila Firmino Gonçalves Pecoli, Anderson da Silva Rosa, Mônica Andrade Lima Gabbay, Sérgio Atala Dib

**Affiliations:** 1 Diabetes and Endocrinology Center, Internal Medicine Department, Universidade Federal de São Paulo, São Paulo 04039-032, Brazil; 2 Nursing Department, Universidade Federal de São Paulo, São Paulo 04039-032, Brazil

**Keywords:** type 1 diabetes mellitus, qualitative research, patient experience, self-management, diabetes care

## Abstract

Type 1 diabetes imposes a complex and challenging routine on patients and caregivers. Therefore, considering individual experiences and personal facilitators to promote assertive interventions is crucial. However, no studies have addressed these perspectives in the Brazilian adult population. We aimed to identify psycho-behavioral characteristics perceived as facilitators for coping with the condition. We used a biographical method to conduct semi-structured, face-to-face, in-depth interviews for each participant. Transcripts were analyzed using inductive thematic analysis. Participants (*n* = 22) were aged 18–57 years (mean: 30.2; standard deviation (SD): 8.7), and the duration since diagnosis was approximately 20.6 years (SD: 4.6). A total of 12 (54.4%) were women, 13 (59.1%) used insulin pumps, 14 (63.6%) had at least a college degree, and 13 (59.1%) had HbA_1C_ (glycated hemoglobin) levels above 58 mmol/mol (7.5%). Five major themes emerged: (1) peer learning, (2) ownership, (3) welcoming experiences, (4) equity, and (5) reframing the path (P.O.W.E.R.). All themes appeared in the lived experiences shared by participants with HbA_1C_ levels below 58 mmol/mol (7.5%). Improved glycemic control can be achieved, and the challenges encountered in diabetes care within similar socioeconomic contexts can be addressed by an interdisciplinary care team that takes P.O.W.E.R. into consideration when providing person-centered care strategies.

## 1. Introduction

Brazil ranks as the third country worldwide in terms of type 1 diabetes cases, with approximately 92,300 children aged 0–14 years old and 476,200 adults over 20 years old diagnosed with this condition. Its estimated annual incidence is 8900 new cases in children and adolescents (0–19 years). Brazil also ranks third in type 1 diabetes-related financial expenses [1]. Owing to its complex socioeconomic environment, accessing the latest technologies and best treatment options for type 1 diabetes can be challenging and lead to negative impacts on the quality of life [2]. These statistics underscore the importance of addressing and promoting appropriate care services from the time of diagnosis to achieve effective outcomes.

Type 1 diabetes entails the establishment of a complex and demanding routine for patients and their caregivers, leading to social, behavioral, and emotional consequences [2]. If the initial experience of managing diabetes is not properly supported, it can trigger ongoing psychological issues that hinder patients’ coping ability and adversely affect their overall health [3,4,5,6]. Therefore, considering individual experiences and facilitators when promoting assertive interventions is crucial [7].

Young people with type 1 diabetes face general life challenges and approach them similarly to their peers without diabetes. They actively participate in their treatment; however, they often do not consider the condition or the treatment-related tasks as the most important stressors in their daily lives [8]. Identifying daily stressors and coping strategies is an important component of support. Coping with stressors is usually facilitated by a good support system that includes parents, family members, friends, healthcare professionals, and school nurses [9].

Age, disease duration, stress perception, and family factors are the greatest predictors of adaptation for children with type 1 diabetes [8]. A systematic and narrative review revealed that the inter-related needs of children following the diagnosis comprise providing adequate time for adjustment, opportunities for engagement and exploration, meaningful participation, and supportive relationships that provide the appropriate protection without differentiation [10].

Lived experiences surrounding diabetes management in adolescents with type 1 diabetes and their parents can be characterized by three distinct stages: adapting to the diagnosis, learning to live with the disease, and achieving independence. Learning through experience is key to developing self-management and independence skills [11].

The literature also demonstrates that the barriers encountered by adults with type 1 diabetes can be mitigated through supportive care from friends, family members, and healthcare professionals. A collaborative approach, treatment guidance, and personalized use of technical devices are crucial resources for nurturing skills and knowledge, facilitating diabetes management, and improving quality of life [12].

An investigation into the experiences of adults with type 1 diabetes with HbA_1C_ (glycated hemoglobin) levels of 53 mmol/mol (7%) or lower revealed that the journey toward effective management involved experimentation, support, knowledge seeking, and trust building, resulting in a sense of security in balancing lifestyle and socioenvironmental factors. Flexibility in self-care techniques and lifestyle behaviors emerged after developing confidence in the treatment and taking responsibility for glucose control. Accepting diabetes as a part of life and committing to its management were key factors in achieving this outcome [13].

Few Brazilian studies have explored the perspectives of children and adolescents with type 1 diabetes regarding treatment facilitators [14,15]; however, there is a gap concerning the adult perspective or considering the influence of socioeconomic and health public services. The adult perspective introduces maturity to understand the perceived challenges of living with this condition. They offer insights into the lessons learned throughout treatment and suggest new strategies and interventions for healthcare professionals and caregivers [16].

To minimize this gap in literature, we aimed to identify the lived experiences shared by Brazilian adults with type 1 diabetes mellitus and the psycho-behavioral characteristics perceived as facilitators for coping with this condition, thereby contributing to a better quality of life. To our knowledge, this is the first study of its kind.

## 2. Materials and Methods

### 2.1. Setting and Sample

The research was conducted between September 2019 and March 2020 at the Diabetes and Endocrinology Center of the Federal University of São Paulo, where approximately 1200 cases of type 1 diabetes mellitus are treated yearly. In most cases, the patients attended their first medical visit over a year after diagnosis.

To ensure a robust theoretical interpretation, participants were sampled from a population with diversity in age groups, treatment regimens, time of diagnosis, and glycemic control. The inclusion criteria were as follows: (a) a confirmed diagnosis of type 1 diabetes for at least 10 years, (b) age 18 years or older, (c) willingness to share personal stories, and (d) absence of severe mental impairment that would interfere with an in-depth interview. Although we did not consider it as an eligibility criterion, none of the participants had prior contact or participated in any psychological care session with the researcher before the interview. Data of the potential participants were verified in their electronic medical records, and an experienced clinical psychologist trained in qualitative research design methods (PP) extended a personal invitation for participation on the day of their medical appointment. Participation was voluntary, and written consent was obtained before each interview. Sampling continued until interviewees’ responses indicated the emergence of recurring themes, and data saturation was achieved.

### 2.2. Procedures

This qualitative study utilized the biographical method [17] and comprised one semi-structured, face-to-face, in-depth interview divided into two sessions with each participant. On average, the interviews lasted 1 h (50–90 min), and they were audiotaped using a digital recorder. All interviews were conducted by one of the researchers (PP) in a private office at the Diabetes and Endocrinology Center. To ensure confidentiality and privacy, only the researcher and the participant were present at the time of interview. In the first interview session, the participants answered the prompt, “Tell me your story about living with type 1 diabetes”. The second session involved exploring and clarifying the shared content. The participants were also asked the following open-ended questions: (1) What challenges do you remember facing throughout your life with diabetes? (2) How did you overcome these challenges? (3) What lived experiences contributed to your improved quality of life with type 1 diabetes?

### 2.3. Data Analysis

An inductive thematic analysis method composed of six steps [18] was used to identify patterns and clusters of meanings within the data (see Table 1).

The initial phase involved organizing the collected data. All interviews were transcribed verbatim using a professional transcription service. The transcripts were cross-checked against the recordings for accuracy and re-identified to guarantee confidentiality using codes that included the unique participant number (P#) and age (A#). These codes are used in the Results section to identify the source of quotations. The content was uploaded to the qualitative data analysis (QDA) software NVivo version 12.6.0 (QSR International). Two researchers conducted data familiarization that started with a free-floating reading and re-reading process for each transcript.

During the initial coding stage, a meticulous reading process was conducted to develop a draft coding framework. Detailed coding rules and definitions were established by two researchers (PP and MG), and adjustments to improve comprehensibility and utility were made and reviewed. The revised framework was used to code two transcripts together and four additional transcripts independently to ensure agreement. After a discussion of minor discrepancies among all research team members, a consensus on the coding framework was reached [19]. The different backgrounds of the researchers contributed to a richer understanding through their distinct perspectives on the data. The remaining 16 transcripts were coded independently.

The themes were refined by collating codes into potential themes while considering the specificity of each theme and continuously verifying their validity and reliability [20]. The generated content was verified by an experienced external qualitative researcher to ensure trustworthiness [21]. All of the research team members discussed minor discrepancies and generated definitions and names for each theme. At the end of the interpretation phase, a cross-comparison of themes and their prevalence within each transcript was conducted. No participant checking was conducted on the data.

### 2.4. Ethics

The study was approved by the Federal University of São Paulo Ethics Committee and was conducted in accordance with the Declaration of Helsinki (reference number IRB: 68037517.7.0000.5505).

## 3. Results

The 22 participants included individuals aged 18–57 years (mean, 30.2 y.o.; standard deviation (SD), 8.7 y.o.) with a duration since diagnosis of approximately 20.6 years (SD, 4.6). A total of 12 (54.4%) were women, 13 (59.1%) used insulin pumps, 14 (63.6%) had at least a college degree, and 13 (59.1%) had HbA_1C_ levels above 58 mmol/mol (7.5%).

Seven (78%) participants with HbA_1C_ levels below 58 mmol/mol (7.5%) used continuous subcutaneous insulin infusion (CSII) without connection to a continuous glucose monitor (CGM). In contrast, the group with HbA_1C_ levels above 58 mmol/mol (7.5%) was homogeneously distributed between CSII users and non-users.

Comparison between the two groups revealed other discrepancies. Participants with HbA_1C_ levels below 58 mmol/mol (7.5%) had a lower incidence of diabetes complications (two with retinopathy (22.2%) and one (11.1%) with neuropathy) and lower use of psychiatric medication (22.2%). Table 2 presents other sociodemographic characteristics and their relationship with HbA_1C_ levels.

Five major themes related to experiences that facilitated coping with the condition and improved quality of life emerged: (1) peer learning, (2) ownership, (3) welcoming experiences, (4) equity, and (5) reframing the path. Table 3 presents the themes, subthemes, and prevalent codes. All five major themes (P.O.W.E.R.) were present in the lived experiences shared by each participant with HbA_1C_ levels below 58 mmol/mol (7.5%).

Table 4 provides more information on the theme distribution among the data collected from each study participant and their correspondence with HbA_1C_ levels.

### 3.1. Peer Learning

This theme covered the experiences shared among peers with diabetes related to diagnosis acceptance, subsequent adjustments, and daily challenges of living with it.

Fifteen participants expressed the importance of discussing and listening to the difficulties associated with self-management. This open communication allowed them to better understand their emotions regarding their health condition. The sense of connection formed through these interactions was recognized as an important factor in facilitating diagnosis acceptance and improving their quality of life.

P1: I realized that I could help people with my story, and, in some way, what they say also makes me reflect on many things I had gone through. For the first time, I opened myself and wanted to know and hear what others were saying. My problem was so big that I did not want to know about other people’s problems. I wanted them to hear mine, but the group was an exchange. Therefore, I saw that I could help people with what I am good at, and what others are good at is often what I am bad at. I thought it was cool to be in this group. (A30)

P7: Each person said what they usually did, what they were afraid of, what they weren’t afraid of when injecting insulin. The room monitor also had diabetes at the time. She sat next to me and said where she was going to inject in my body and said that I could do it where I felt better. She explained tricks to inject insulin. On that day I injected it alone. I thought it was great, because I saw that it did not hurt at all and sometimes when my mother injected it, it hurt. (A29)

P21: We worked a lot on fear, the importance of care, and exchange of experiences. Then, I started attending a group for teenagers. The teenager’s group was much more interesting because the children group had mothers present; however, only teenagers and older people were present in the teenagers group. Back then, I was a 14-year-old kid. There was a man in his twenties who traveled the world on a trail, doing a lot of things. Look, that is cool. The man had diabetes, just like me. (A37)

### 3.2. Ownership

This theme covered the experience of taking responsibility for self-management and diabetes care. Sixteen participants emphasized that engagement with their treatment was promoted by accepting diabetes as an integral part of themselves and recognizing its management as essential to achieving their personal life goals.

P11: This is more ours than anyone else’s. It is not your mother who will take care of you, nor your husband; it is you. If you are unaware of this, you will not achieve anything in life; you will be unhealthy, and that is the most important thing. (A29)

P6: Exactly, to be a mother. It is my dream, and that is what rules what I think. If I do not take care of myself, how can I have a peaceful pregnancy and have my child? Understood? That is what moves me. It may be that I do not even have a child. I do not know, but that triggered me to change and made me think more about my body and myself. (A28)

P14: I think this has more to do with a kind of maturity than with someone telling you to. When I was a child, it was not me who managed my life. I obeyed what my mother said. Today, I am the one who manages my life. I am the one who must check my schedule–what fits and what does not fit. When I was a little younger, I was distressed because I could not manage everything I had to do, and today I can see that even if I am unable to make time for everything, what I would have to do in the ideal world, I can do what is possible and do my best to get well. (A27)

Fourteen participants considered the experience of overcoming the fear of complications as another component that contributed to their engagement in self-care and improving their quality of life. It became apparent mainly when diabetic complications appeared and threatened their achievements. Establishing a balance between control and fear was achieved by recognizing the possibility of losing and the desire to avoid giving up on their goals. This triggered a sense of ownership that reflected how they treated their bodies, lives, and diabetes education.

P7: I was 15 when I discovered retinopathy and started appropriate treatment. I finally understood what glycated hemoglobin was and why I had to write a lot of things in the diary. Until then, we had just written it down, and I did not understand why I had to write it all down. (A29)

P14: At the last visit I came to, I brought this test result and then they said that the most normal thing was to give diabetic retinopathy and not cataracts; however, regardless of what they said, I was terrified. I said, how am I going to be a doctor if I cannot see? There is a whole future ahead of me. Then I started. I firmly did what I had to do. I did it. (A27)

P17: I have just become a father. My son is so little, and I want to enjoy life with him. So, I have to act preventively to avoid complications. I have to take care of myself so that I can guide him and see him growing too. (A26)

### 3.3. Welcoming Experiences

This theme explored the positive and supportive approaches encountered in diabetes care. These experiences were primarily present in the interactions with peers who did not have diabetes, family members, and healthcare professionals. Seventeen participants emphasized empathy as an important contributor to welcoming experiences. Empathy was evident when others embraced a person-centered care model in an attempt to contextualize their beliefs, values, worries, and challenging situations. Empathic gestures fostered unarmed relationships with caregivers and eased the process of requesting help when needed.

P6: The idea of trying to understand what is happening, why the blood glucose is high, and what happened behind all this. It is not that… ‘oh, it is high, you have to change, or you did something wrong’. No, but ‘why did you do something? What happened that day?’ This is really the sense of feeling welcomed because you are trying to understand why it went wrong, and blood glucose is not something you put in a jar; it stays there quietly. (A28)

P4: For those who do not know, I usually explain these issues. Therefore, they end up being more careful and concerned with me. I see it as something good because I have it when I need it. I know that I will have someone to help me. (A28)

P5: What helped me a lot were people. Specific people. I was lucky to have them in my life. My brother has always been interested, not in my diabetes, but in me. Hence, diabetes consequently became his interest. He is always worried about my feelings, how I am, and what I need. (A27)

Furthermore, 13 participants felt welcome when they could establish a partnership with someone who shared the responsibility of managing their prescribed conditions to achieve treatment goals. Concerns about food, hypoglycemia episodes, and the best treatment options exemplified the collaborative support that encouraged them to view diabetes as a manageable condition and reassured them that they were not alone in its management.

P8: She was always there. She worked a lot, but stayed with me at the hospital. She always helped me. When I lived with them, she always cooked for me; she tried to do it in the best way possible. (A25)

P21: My family always helped a lot. So, when it comes to diabetes, this type of support is essential. Looking for alternatives for food, participating in groups, and looking for better treatments. They were always there. (A37)

P21: It is always good to have someone on your side, someone who can help you, especially when you have diabetes. Like my roommates who helped me a lot. During severe hypoglycemic events, they already knew what to do. They already knew every protocol that had to be done. (A37)

### 3.4. Equity

This theme covered the experiences of encountering inclusivity, fairness, and equal opportunities in which diabetes did not lead to exclusion from various social groups or cause harm to individuals. Twelve participants recognized the importance of being part of accepting and diverse communities that positively embraced the presence of diabetes in their lives.

Equity was fostered by embracing diversity and played a vital role in accepting the diagnosis and its implications on self-management.

P7: My mother always let me attend every field trip. She always let me do everything that a child could do. Of course, she was a little scared, but she let me enjoy all the opportunities I had as a normal kid. (A29)

P8: I think what was important for me was not to be seen as different. I am like anyone else. Yes, I must take insulin, I have to measure blood glucose, but it does not make myself different from other people. This is really important, to put myself into everyday life like the normal person that I am. I take this as a demonstration of affection when people consider me this way and try to understand what diabetes really is. (A25)

P19: I accepted the disease when I started college. By that time, I had started to live with people who were different from me and lived well, but not because of the disease. They were different people. I started to see that diabetes was not a problem. (A34)

P21: It was interesting and helped me a lot to have a network of friends who did not exclude me or make me feel different within the group. I think that was excellent and crucial because it was not something like: “Oh, we will not be able to do this because P21 is with us”. No, I never had that. We have always done things together, and I have never felt different from them. I was always part of the group. (A37)

### 3.5. Reframing the Path

This theme covered the experiences of re-evaluating variables and meanings related to diabetes, leading to new perspectives on self-management and the condition. Fourteen participants considered the possibility of finding and choosing treatment alternatives through online information and conversations with healthcare professionals as facilitators of coping with diabetes. This opened new paths, allowing them to reframe their understanding of the treatment and chronic conditions.

P8: I think it was important for me to go further, ask, understand what it was, try to see it within my routine and see other people, and see that life was normal because people tried to show that it was not. ‘Oh, poor thing’. No, it is not poor. It is a disease that has control; I can have a normal life. Why do I know that? I read a lot and I saw other people in the same condition, even people younger than me. This was very remarkable and I used this opportunity to really know the alternatives. (A25)

P21: I think one of the things that changed the most for me was breaking of several myths such as forbidden food. It is not that you are prohibited to eat, it is preferred to be avoided, but you can eat to some extent if you do the carb counting which I was a novelty for me that helped a lot to be more flexible with diabetes. (A37)

Additionally, 14 participants acknowledged the value of reflecting on the aspects surrounding their diagnosis and the journey on which they have embarked throughout their treatment. By recognizing and acknowledging their emotions and experiences, they could change their perspectives and behaviors toward diabetes.

P8: I looked through everything that had happened, remembering all the stages of the diagnosis, everything that was so difficult and realized the path I was taking and where it would lead me. It was really an act of reflection, and I went back to doing it a little more carefully. (A25)

P10: I went through everything. All of these were caused by poor control. I understood that I needed to take care of myself to be different. I think this helped me understand and clarify my path. It is one thing when the doctor says it, but you do not know him. Another thing is that you are living as the person who suffers the consequences and sees what happens. (A35)

## 4. Discussion

This study identified the lived experiences shared by Brazilian adults with type 1 diabetes mellitus and the psycho-behavioral characteristics perceived as facilitators for coping with the condition, thereby contributing to a better quality of life. Peer learning, ownership, welcoming experiences, equity, and reframing the path were the five major themes that emerged from the collected data. Good metabolic control, adequate therapeutic adherence, and psycho-social and emotional well-being have a positive impact on quality of life [22]; however, our data revealed that even individuals with better glycemic control, awareness of self-care importance, or access to better insulin, innovative monitoring, and glycemic correction devices face emotional struggles related to diabetes management that impact their mental health.

To the best of our knowledge, this is the first Brazilian qualitative study to explore this content in this specific population. We believe the implications of our research extend beyond the immediate scope of the studied population of Brazilian adults with type 1 diabetes mellitus. By understanding the lived experiences and the facilitators for coping, healthcare professionals can better tailor their interventions and support systems to address the unique challenges and needs of those with type 1 diabetes.

Peer learning emerged as a great opportunity for individuals to connect with the discomfort and challenges associated with diabetes. Learning from and engaging in conversations with others with similar life experiences enabled them to gain a broader perspective on what living with diabetes entails, relativize the challenges, and alleviate feelings of isolation. Peer support was facilitated through diabetes associations, summer camps, and online peer-to-peer support groups. According to a systematic review, evidence on the effectiveness of peer support in diabetes outcomes is limited and inconsistent [23]; however, our findings are consistent with recent literature and reveal that through peer relationships, participants could validate their difficulties, reframe their understanding of failures, and develop new, concrete plans based on tangible parameters [16,24].

Consistent with previous studies, the sense of ownership emphasizes the importance of considering diabetes and its management as purposeful and linked to individual objectives [13,16,25]. The fear of complications appeared to motivate self-care; its potential effect on personal achievements and the belief in the possibility of reversibility through self-management changes empowered individuals. Establishing a balance between control and fear is important in self-management to obtain positive results [26,27]. Successful management of type 1 diabetes is supported by interventions that promote acceptance of the diagnosis and foster confidence through experiment learning such as trial and error [10,13].

Empathy emerged as a key factor in creating a welcoming environment for people with diabetes. Several studies have demonstrated the importance of empathic relationships between people with diabetes and caregivers [28]. Thirty years ago, the connection between physician empathy and the clinical outcomes of this population was demonstrated [29]. Collaboration between healthcare professionals was associated with better self-reported clinical results [30]. Thus, healthcare professionals’ knowledge of diabetes alone is insufficient to be effective in their care. Action should be taken to empower healthcare professionals through psycho-social training to enable them to explore patients’ perspectives undergoing treatment. This step is crucial in acknowledging the impact of chronic conditions on stakeholders and promoting respectful communication, cultural competency, and empathy [31].

Collaborative support is a key element that can enhance self-management. Complex decision making and cooperation among patients, caregivers, and social support systems are essential to obtain the best results. People living with chronic conditions such as diabetes cope with stressors within an interpersonal context and are not isolated from each other. The dyadic nature of collaboration and the experiences acquired through the interactions established with couples and family members throughout treatment are important aspects to consider when designing and improving interventions [32]. Involving the person with diabetes and the caregiver in a joint effort to solve problems related to diabetes management can be mutually beneficial in activating the support process and alleviating distress [32,33]. Collaborative parent–child relationships with high levels of family cohesion, a participatory parenting style, parental monitoring, division of responsibilities for managing diabetes, and collaborative solutions to face difficulties are related to better treatment adherence and glycemic control [34]. Metabolic control is not correlated with perceived support; however, early discontinuation of support can compromise diabetes management [35]. Caregivers play a crucial role in encouraging children to be more proactive in diabetes discussions by allowing them to express their opinions and concerns, share information, and provide solicited guidance. Hence, healthcare professionals should be aware of the transition of care processes from the beginning of the treatment for children and adolescents, as it can impact autonomy and self-management [36].

The sense of equity established through inclusive relationships with peers and family without diabetes contributed to accepting the diagnosis and its implications for self-management. Receiving the same opportunities and addressing the differences as avoidable and remediable expanded their perspectives on what is considered normal or healthy. This stretched their capacity to make individual choices, favoring personal interests and development. The literature emphasizes the importance of receiving compassionate and caring psycho-social support from family, friends, healthcare professionals, and other people with diabetes [10,37]. One in five people with this condition experienced discrimination because of their diagnosis [38]. The feeling of being different is a major challenge in living with diabetes [35,37], and being defined by it may influence engagement in self-management in the presence of peers [16]. Healthcare professionals should work toward the destigmatization of diabetes and consider the psychological aspects and experiences of each patient throughout treatment to design and improve effective interventions [39].

Our findings highlight the importance of reframing individual paths toward diabetes. The literature reveals the complex learning process of living with this chronic condition [7,16]. The duration of the diagnosis is not relevant to its development [40]. Diabetes can considerably disrupt future life plans and generate emotional distress [7]. Illness perception directly influences self-efficacy, maladaptive coping, quality of life, and anxiety symptoms [41]. As individuals attempt to integrate the disease into their daily lives, an inner dialogue between the self, body, and life is ongoing [40]. To prevent continuous psychological problems leading to suboptimal self-management behaviors, close attention should be given to the initial treatment experiences [10,25]. When accessible and sensitive to their needs, healthcare professionals can recognize the personal emotional impact of diabetes and offer appropriate support information to help individuals find the best treatment alternatives [25].

We believe our findings might be potentially applicable to other chronic conditions. Healthcare providers can leverage these insights to develop tailored and comprehensive care plans that address the emotional well-being and self-management needs of patients living with chronic illnesses. Moreover, by recognizing the universality of these coping mechanisms, healthcare professionals can foster a more empathetic and inclusive approach to patient care, acknowledging that individuals with different conditions may share similar emotional experiences and coping challenges.

Seven participants (78%) with HbA_1C_ levels below 58 mmol/mol (7.5%) used CSII without using a CGM and received diabetes care through a multidisciplinary educational program. In contrast, in the group with HbA_1C_ levels above 58 mmol/mol (7.5%), the distribution of CSII users and non-users and participation in educational programs was more homogeneous. The literature reveals that CSII and multiple daily injections have similar effects on glycemic control and hypoglycemia, with CSII having a favorable effect on glycemic control in adults with type 1 diabetes [42]. Few studies have demonstrated the efficacy of educational groups on CSII users; however, providing basic education and re-education for experienced type 1 diabetes CSII users benefits the therapy and decreases HbA_1C_ levels [43].

Our study data also revealed a higher incidence of diabetes complications and the use of psychiatric medication in participants with higher HbA_1C_ levels, in line with previous studies [44,45,46]. For individuals with type 1 diabetes, poor glycemic control history is associated with an increased risk of anxiety- and mood-associated disorders [47]. Further, one of the main predictors of poor quality of life in patients with type 1 diabetes is the presence of comorbidities and complications [48,49].

One strength of this research is the diverse perspectives brought by the participants, which allowed us to thoroughly explore the challenging aspects of living with diabetes. Furthermore, the emergent themes align with existing literature and recent clinical consensus.

Our research had some limitations inherent to the methodology used. The small sample size prevented us from establishing the statistical relevance of the correlation between HbA_1C_ levels and the sociodemographic characteristics of the study participants related to insulin administration, diabetes complications, and educational and mental health approaches. However, we recognize that these factors may have influenced the study results. The absence of triangulation with perspectives of people with diabetes of different age ranges, caregivers, and healthcare professionals restricted our ability to fully generalize the findings to others with similar conditions. The limited geographic scope of the study, which was confined to a specific diabetes center, may also restrict the generalizability to a broader demographic of individuals with type 1 diabetes across different regions in Brazil.

Furthermore, the use of purposive sampling to recruit participants from a specific clinical setting introduced potential selection bias, as the participants may not have fully represented the diverse experiences of all Brazilian adults with type 1 diabetes. The presence of recall bias is another concern, as participants were required to recall past experiences, which could lead to inaccuracies or inconsistencies in their responses. Moreover, the absence of a comparison group in the study prevented insights and comparisons between different subgroups, limiting the understanding of how various characteristics might vary across distinct populations.

To address these limitations and improve the generalizability of our findings, further studies implementing next-generation approaches are recommended [50]. Future research could employ a larger and more diverse sample of participants encompassing individuals from various regions and healthcare settings. Employing a triangulation approach with multiple data sources or methodologies could strengthen the validity of the results. Additionally, researchers could adopt a comparative design to investigate and compare the perceived facilitators among different subgroups, providing a more comprehensive understanding of coping strategies for type 1 diabetes. Moreover, using other sampling techniques, such as random sampling, could help reduce potential selection bias and enhance the representativeness of the findings. Despite these limitations, the present study contributes valuable insights into the psycho-behavioral characteristics that facilitate coping with type 1 diabetes among Brazilian adults, thus serving as a foundation for future research in this area.

One hundred years after the discovery of insulin, advancements have been made in diabetes treatment, particularly in the development of new monitoring and glycemic correction devices. However, these innovations will only be effective if people use them [51]. This highlights the importance of providing access to new technologies, which undoubtedly improve the lives of individuals with diabetes, and the need for comprehensive training that addresses human behavior and psycho-social factors that influence self-management.

## 5. Conclusions

All participants with HbA_1C_ levels below 58 mmol/mol (7.5%) viewed the experiences related to the five emergent themes (P.O.W.E.R.) as facilitators. This reinforced the importance of implementing person-centered care strategies throughout treatment. These strategies should be delivered by an interdisciplinary healthcare team and include psycho-social assessment [25,52]. Such an approach can foster a comprehensive understanding of living with type 1 diabetes and empower individuals to navigate their paths of self-care and self-management.

However, this remains a distant reality for most Brazilians undergoing diabetes treatment, particularly in public healthcare settings. Additionally, the scarcity of psychologists specialized in this chronic condition further complicates the integration of these practices into the country’s healthcare services.

Based on our findings, we can conclude that improved glycemic control outcomes and addressing the challenges and struggles associated with diabetes care in similar socioeconomic contexts can benefit from combined person-centered care strategies. These strategies should be delivered by an interdisciplinary care team that considers the elements of P.O.W.E.R. The interactions established between peers, caregivers, healthcare professionals, and individuals with diabetes can promote the acceptance of self-management as a valuable factor in achieving personal goals and enjoying a healthier life.

## Figures and Tables

**Table 1:** Six-step framework used to identify patterns or themes within qualitative data in inductive thematic data analysis (adapted from Braun & Clarke’s *Handbook of Research Methods in Health Social Sciences* [18]).

Phase	Step	Activity (Objective)
Organization	Become familiar with the data	Professional verbatim transcription Recording accuracy check Re-identifications NVivo collected data upload
		Free-floating reading and re-reading of the transcripts case-by-case Noting initial ideas
Exploration	Generate initial codes	Coding elements of interest in the data Collating data relevant to each code Draft coding framework—establishment of detailed coding rules and definitions Adjustments to improve comprehensibility and utility Discussion of minor discrepancies by all research team members Agreement on coding framework
	Search for themes	Collating codes into potential themes and gathering all data relevant to each potential theme
Interpretation	Review themes Define themes	Ongoing analysis to refine the specifics of each theme Reviewing themes and checking validity and reliability Generated content verification by an external experienced qualitative researcher Discussion of minor discrepancies Generating definitions and names of each theme
Report	Writing-up	Cross-comparison of themes and their weight within each transcript
		Presentation of the research findings

**Table 2:** Sociodemographic characteristics of study participants (*n* = 22).

	HbA_1C_ Levels	
Characteristics	<58 mmol/mol (7.5%)	>58 mmol/mol (7.5%)
	*n* = 9	*n* = 13
	Frequency (%)	Frequency (%)
Age (years)		
Under 25	3 (33.3)	3 (23.1)
26–40	6 (66.7)	8 (61.5)
Above 40	0 (0.0)	2 (15.4)
Women	6 (66.7)	6 (46.2)
Educational status		
Elementary	0 (0.0)	5 (38.5)
High school	1 (11.1)	2 (15.4)
College degree	5 (55.6)	6 (46.2)
Postgraduate	3 (33.3)	0 (0.0)
Employment	9 (100.0)	11 (84.6)
Treatment type		
CSII	7 (77.8)	6 (46.2)
Non-CSII	2 (22.2)	7 (53.8)
Diabetes duration in years		
11–20	4 (44.4)	7 (53.8)
Above 21	5 (55.6)	6 (46.2)
Conditions at diagnosis		
Hospitalization	6 (66.7)	10 (76.9)
Ketoacidosis	3 (33.3)	10 (76.9)
Hospitalizations along treatment		
Ketoacidosis	3 (33.3)	4 (30.8)
Severe hypoglycemia	4 (44.4)	8 (61.5)
Mental health support		
Psychotherapy	4 (44.4)	7 (53.8)
Psychiatric medication	2 (22.2)	6 (46.2)
Diabetes education support		
Diabetes summer camps	4 (44.4)	2 (15.4)
Educational groups	9 (100.0)	9 (69.2)
Complications		
Retinopathy	2 (22.2)	8 (61.5)
Neuropathy	1 (11.0)	3 (23.1)
Nephropathy	0 (0.0)	0 (0.0)

CSII, continuous subcutaneous insulin infusion; HbA_1_c, glycated hemoglobin.

**Table 3:** Themes, subthemes, and prevalent codes that emerged from the interviews of study participants.

Themes	Subthemes	Prevalent Codes
Peer learning	Peer exchange	Learning the daily challenges of peers with T1DM Hearing diagnosis acceptance testimonials by peers with T1DM
	Connection	Sense of belonging to a group by sharing diabetes management experiences Feeling connected to a person with diabetes by sharing common struggles, emotions, and sensations
Ownership	Purpose	Accepting diabetes as an inseparable part of themselves (“This is more ours than anyone else’s”) Establishing a partnership with diabetes management as an essential component to succeed in their personal life goals
	Overcoming fear of complications	Establishing a balance between control and fear to obtain positive results toward self-management after the diagnosis of a diabetes complication Establishing a vision for the future after realizing the desire of not giving up on personal goals after the diagnosis of a diabetes complication (“There is a whole future ahead of me”)
Welcoming experiences	Empathy	Feeling listened to and understood in their struggles with diabetes care Feeling that their beliefs and values were considered in diabetes management strategies
	Collaborative support	Partnering up with someone who would take care of the prescribed conditions to achieve treatment goals together (“They were always there”) Receiving support that encouraged them to view diabetes as a manageable condition
Equity	Equal opportunities	Being provided with equal conditions in life that protected them from discrimination Being assured of making full use of the opportunities offered and fulfilling their potential
	Diversity	Feeling recognized for their different background and experiences in a non-diabetes-related social environment Feeling diabetes did not exclude them from belonging to social groups
Reframing the path	Finding alternatives	Identifying treatment alternatives for solving issues related to diabetes through online information Identifying treatment alternatives for solving problems related to diabetes through healthcare professionals
	Reflecting on diabetes	Reflecting on their feelings regarding the aspects surrounding the diagnosis Realizing the path they have tread during the treatment

T1DM, type 1 diabetes mellitus.

**Table 4:** Distribution of themes and subthemes in the collected data of each study participant and their correspondence with HbA_1C_ levels (*n* = 22).

			Themes and Subthemes									
			Peer Learning		Ownership		Welcoming Experiences		Equity		Reframing the Path	
Participant	Age	HbA_1C_	Peer Exchange	Connection	Purpose	Overcoming Fear of Complications	Empathy	Collaborative support	Equal Opportunities	Diversity	Finding Alternatives	Reflecting on Diabetes
P1	30	8.8	2	7	1	5	4	0	0	0	0	4
P2	27	8.6	0	0	6	1	6	0	0	2	3	1
P3	18	9.6	0	0	0	0	0	0	0	1	0	2
P4	28	12.7	0	2	0	0	4	7	1	0	2	2
P5	27	9.6	1	2	0	0	5	0	0	0	3	0
P6	28	6.8	3	3	4	3	13	0	1	1	1	4
P7	29	6.9	4	10	4	4	6	1	2	4	2	3
P8	25	7.2	2	4	1	1	6	1	1	3	2	12
P9	37	6.9	1	3	4	0	1	0	1	1	5	1
P10	35	6.4	2	2	2	0	6	1	2	3	2	3
P11	29	7.4	3	9	2	1	1	1	1	2	1	0
P12	22	11.0	0	0	0	1	1	0	0	0	0	0
P13	28	10.0	0	0	0	2	0	0	0	0	0	1
P14	27	9.1	1	2	6	4	1	3	0	0	0	0
P15	48	9.6	1	2	0	0	0	1	1	1	2	0
P16	30	8.0	0	0	2	0	0	1	0	0	0	0
P17	58	7.7	0	0	5	1	3	1	0	0	0	0
P18	33	6.9	1	1	2	0	6	2	1	3	1	2
P19	34	6.1	1	2	1	1	2	0	2	6	3	4
P20	20	8.1	0	0	2	1	2	4	0	0	1	1
P21	37	6.3	3	6	3	2	1	3	4	5	5	2
P22	25	8.4	1	2	3	5	0	3	0	0	0	0
Total pieces of data			26	57	57	32	68	29	17	32	33	42

HbA_1_c, glycated hemoglobin.

## Data Availability

The data are unavailable due to privacy or ethical restrictions.
